# Construction and Validation of the 17-Item Stroke-Specific Quality of Life Scale (SS-QOL-17): A Comprehensive Short Scale to Assess the Functional, Psychosocial, and Therapeutic Factors of QOL among Stroke Survivors

**DOI:** 10.3390/ijerph192315668

**Published:** 2022-11-25

**Authors:** Fouad Sakr, Mariam Dabbous, Marwan Akel, Pascale Salameh, Hassan Hosseini

**Affiliations:** 1 École Doctorale Sciences de la Vie et de la Santé, Université Paris-Est Créteil, 94010 Créteil, France; 2 UMR 955 INSERM, Institut Mondor de Recherche Biomédicale, Université Paris-Est Créteil, 94010 Créteil, France; 3 School of Pharmacy, Lebanese International University, Beirut 1105, Lebanon; 4 International Pharmaceutical Federation (FIP), 2517 The Hague, The Netherlands; 5 INSPECT-LB: Institut National de Santé Publique, Épidémiologie Clinique et Toxicologie-Liban, Beirut 1103, Lebanon; 6 School of Medicine, Lebanese American University, Byblos 4504, Lebanon; 7 Faculty of Public Health, Lebanese University, Beirut 1103, Lebanon; 8 Department of Primary Care and Population Health, University of Nicosia Medical School, Nicosia 2408, Cyprus; 9 Stroke Unit, Service de Neurologie, CHU Henri Mondor, 94010 Créteil, France

**Keywords:** stroke, quality of life, validated scale, stroke survivors, SS-QOL, stroke outcomes, short scale, comprehensive scale

## Abstract

(1) Background: The exiting stroke-specific quality of life (SS-QOL) measure scales are limited by their excessive length, inconsistent validity, and restricted breadths of assessment. The objectives of this study were to construct and validate a comprehensive short SS-QOL scale to assess stroke-related outcomes and QOL and determine the socioeconomic, sociodemographic, and pharmacotherapeutic predictors of QOL among stroke survivors. (2) Methods: The novel 17-item SS-QOL scale (SS-QOL-17) was constructed with the aim of providing a well-balanced measuring tool to depict QOL widely while ensuring the simplicity of administration. (3) Results: The SS-QOL-17 structure was validated over a solution of three factors with a Kaiser–Meyer–Olkin measure of sampling adequacy = 0.894 and a significant Bartlett’s test of sphericity (*p* < 0.001). The Cronbach’s alpha of the SS-QOL-17 was 0.903. Better QOL was correlated to financial wellbeing (beta 0.093, *p* < 0.001), and medication adherence (beta 0.305, *p* = 0.004), whereas reduced QOL was correlated to older age (beta −0.117, *p* = 0.014), illiteracy (beta −6.428, *p* < 0.001), unemployment (beta −6.170, *p* < 0.001), and higher amount of prescribed medication (beta −1.148, *p* < 0.001). (4) Conclusions: The SS-QOL-17 is a valid and reliable tool with promising psychometric properties. It is useful in clinical practice and research settings to evaluate the post-stroke therapeutic and rehabilitation outcomes.

## 1. Introduction

Stroke is a leading cause of worldwide disability, morbidity, and mortality [1,2]. It poses a significant impact on the quality of life (QOL) of the survivors, who typically continue to experience long-term sequelae [3]. Post-stroke complications include functional, mental, social, and emotional difficulties to lead a satisfactory life [4,5]. The indicators of functional outcomes are crucial in post-stroke care and rehabilitation. Nonetheless, the assessment of disability and impairment is insufficient alone to determine the true stroke impact [6]. Health-related quality of life (HRQOL) is being progressively employed to evaluate the situation of stroke survivors [6]. Indeed, the assessment of HRQOL is getting more prominent and widely acknowledged for chronic medical conditions as stroke. This assessment measures aspects that patients value and considers how the health situation of patients affects their capacity to live satisfactorily [7].

QOL is perceived differently between patients based on their own aspirations, concerns, and objectives, as well as their cultural background and value systems [8]. Depicting the consequences of stroke and assessing the therapeutic results have been the two major goals of measuring stroke outcomes [9,10]. The most often-employed HRQOL measures are generic, which means that they were created to determine the effects of typical health conditions on daily living in the general population. The EuroQol-5D (EQ-5D), Short Form-6D (which was created from the Short Form-36), and Health Utilities Index are the most well known of these generic utility measures [11,12,13]. Although all have been utilized in stroke, some even extensively, a principal aspect and conceivable inadequacy of these measures is that they assess the health profile domains that are valued by the general population rather than stroke patients [14]. The use of generic tools for the evaluation of QOL that relate to stroke are no longer suitable due to the developing research that shows that patient preferences for health states differ significantly from those of the general population [15]. Therefore, there is a great demand for QOL utility measures that are stroke-specific.

The Stroke-Specific Quality of Life Scale (SS-QOL) is the first HRQOL utility measure for stroke survivors [16]. It was created in 1999 to quantify the impacts of stroke and assess the outcomes of treatment strategies. The SS-QOL is a long 49-item stroke-specific tool that provides a total QOL score and 12 subscale scores relating to mobility, cognition, mood, functionality, and social roles [17]. Nevertheless, previous studies have produced one-, two-, four-, eight-, and twelve-domain solutions for the SS-QOL structure [18,19,20,21]. The proposed structure of 12 domains was consequently not supported by these paradoxical construct validity findings, giving clinicians and researchers the option to employ different subscale scores or only the overall score. A further limitation of the SS-QOL is its excessive length, making it impractical for routine use in research and clinical practice owning to the burden of administration.

Abbreviated scales were suggested to provide a balanced wide-range assessment while reducing the length of the measurement. The short 12-item form of the SS-QOL (SS-QOL-12) was proposed by Post and colleagues to assess a 2-factor model of the long SS-QOL Dutch version relating to the physical and psychosocial domains [22]. The main compromise of the SS-QOL-12 is that its physical factor does not assess the performance required for daily functions such as feeding, dressing, grooming, and toileting. Similarly, the psychosocial factor does not include any items related to irritability and family engagement. The short form of the Stroke Impact Scale (SF-SIS) is another abbreviated scale that includes eight items to assess functional and QOL measures [23]. The SF-SIS is limited by its breadths of assessment, as daily, family, and psychosocial functions are not explicitly measured.

Stroke-related complications and their consequences on the QOL domains are reportedly correlated with treatment efficacy and persistence [24]. The assessment of therapeutic adherence is therefore suggested to be integral of any stroke-related QOL measurement. This measurement involves multiple factors that could be linked to the ability and willingness of patients to follow a specific course of therapy, which could then illustrate a therapeutic domain of their HRQOL [24].

The available short and validated stroke-related QOL measuring scales might ensure simplicity of assessment but surely compromise the provision of a comprehensive profile of health aspects. Moreover, available scales aim to evaluate the HRQOL after the treatment of a stroke, yet none of these scales incorporates a therapeutic domain within the assessment. Therefore, the development of an abbreviated but comprehensive measuring tool remains warranted to describe broader functional and psychosocial domains, and possibly integrate therapeutic factors that could depict stroke-related outcomes. This study aimed to construct and validate a comprehensive short SS-QOL scale to assess stroke-related outcomes and QOL. It also aimed to determine the socioeconomic, sociodemographic, and pharmacotherapeutic predictors of QOL among stroke survivors.

## 2. Materials and Methods

### 2.1. Construction of the 17-Item Stroke-Specific QOL Scale (SS-QOL-17)

The SS-QOL-17 was constructed after a comprehensive PubMed and Scopus literature review. It included a total of 17 items. The first six items were inspired from the Barthel Index (BI) to assess the daily performance of patients on feeding, bathing, grooming, dressing, toileting, and mobility [25]. The responses to these items were scored on a 2-, 3-, and 4-grade scales to determine the level of dependence in performing daily activities. The next two items were added to the SS-QOL-17 from the Lebanese Medication Adherence Scale (LMAS-14) to assess medication forgetfulness and unwillingness to receive therapy particularly due to cost [24]. Responses to these two items were scored on a four-grade scale ranging from “never” to “always”. Finally, the SS-QOL-17 included 9 items that were inspired from the long 49-item list of the Arabic version of SS-QOL (SSQOL-A). These items aimed to assess general areas of QOL linked to energy, family engagement, speech troubles, future discouragement, mood, cognition, social engagement, and general performance [26]. Responses to the nine items were scored on a five-grade scale ranging from “strongly agree” to “strongly disagree”. The total score of the final SS-QOL-17 was then computed by adding up all responses. The score could range from 17 to 70, with higher scores indicating better QOL.

The aim of development of the SS-QOL-17 was to provide a shortened compressive evaluation of post-stroke QOL in alignment with physical and functional characteristics, therapeutic adherence, and psychosocial features of patients. The final scale was reviewed and verified by two external neurologists to ensure the consistency and adequacy of the scale’s items. All necessary translations to the Arabic language were carried by forward–backward translation and conflicts were resolved by consensus. The conceptual equivalence of the translated materials was reviewed and verified by a language expert.

### 2.2. Study Design and Participants

This was a cross-sectional study that involved stroke survivors with a history of any stroke episode. Stroke survivors were described as post-stroke patients who had been admitted to the hospital for an acute cerebrovascular event, and were discharged alive regardless of the degree of sequelae [27]. Participants were enrolled from pharmacies all over the Lebanese community. The medical profiles of the pharmacies were initially screened for stroke patients. All patients and caregivers who came into the pharmacies to receive prescription therapies that may be used for secondary stroke prevention or that can possibly manage stroke risk factors such as diabetes mellitus, dyslipidemia, cardiac arrhythmia, and hypertension were also screened [28]. Adult patients aged 18 years and above who had ever been diagnosed with a hemorrhagic or ischemic stroke by a neurologist were eligible. Patients having a history of stroke symptoms without previous hospitalization or evident diagnosis were excluded.

Well-trained healthcare professionals conducted phone or in-person interviews with the included patients. When a patient had a communication disability, the immediate caregiver was questioned on the patient’s behalf. The interview began with a brief introduction outlining the study goals and the significance of its outcomes on the QOL of post-stroke patients. Data were collected between October 2021 and June 2022 using a well-structured questionnaire in Arabic, the native language of Lebanon. The interview took around 20 min to complete, including the time to record responses.

### 2.3. Variables and Outcomes

The study questionnaire was composed of three sections. The first section asked questions regarding the sociodemographic and socioeconomic characteristics of the patients such as gender, age, social history, residence area, education level, work status, household income, financial wellbeing, medical coverage, and difficulties to obtain prescribed medications. Financial wellbeing was evaluated by the InCharge Financial Distress/Financial Well-Being (IFDFW) scale, which is a validated eight-item scale that assess financial wellbeing on a scale ranging from 1 to 10. Better financial wellbeing is indicated by higher scores [29]. The scale’s Cronbach’s alpha in our sample was 0.936.

The second section of the questionnaire asked questions regarding the patients’ present clinical characteristics, medication use and adherence, past medical history, and stroke history. Adherence to post-stroke medications was assessed by the LMAS-14, which is a generic 14-item scale that was validated to determine adherence to pharmacotherapy in stroke patients [24]. The LMAS-14 items assess adherence on a scale ranging from “never” to “always”, with better medication adherence indicated by greater values of the total score [24]. The Cronbach’s alpha of the scale among our sample was 0.928.

The third section incorporated validated scales that evaluate stroke prognosis, daily activities and performance, and QOL. The modified Rankin Scale (mRS) was used to evaluate stroke outcomes and prognosis on hospital discharge. mRS rates stroke outcomes on a single seven-score scale that ranges from no symptoms (least severe) to death (most severe). Poor prognosis is indicated by dichotomizing the score of the scale at a cut-off point of 3 and above [30]. The BI was also incorporated into this section. BI is a simple prognostic post-stroke tool that determines the current post-stroke daily performance and activities. It assesses 10 functional daily tasks and score the level of independence in feeding, dressing, grooming, bathing, transfers, mobility, stairs, and toileting. Scores of 0–20 indicate total dependency, 21–60 indicate severe dependency, 61–90 indicate moderate dependency, and 91–99 indicates slight dependency [25]. The Cronbach’s alpha among our sample was 0.956. Finally, the SSQOL-A was included, which is a validated Arabic version of the long SS-QOL scale to assess the same 49 items and 12-domain subscales. The SSQOL-A could also have a minimum score of 49 and a maximum score of 245, with higher scores indicating better QOL [26]. The Cronbach’s alpha in our sample was 0.979.

The study outcomes were to (1) construct and validate the novel SS-QOL-17 as a comprehensive yet concise tool to assess QOL among stroke survivors; and (2) determine the socioeconomic, sociodemographic, and pharmacotherapeutic predictors of QOL after a stroke.

### 2.4. Ethical Aspects

The study protocol was approved by the Ethics and Research Committee of the School of Pharmacy in the Lebanese International University (protocol number: 2020RC-048-LIUSOP). The need for written informed consent was waived by the committee because the study, being observational, had no clinical interventions. The confidentiality and anonymity of all participants were guaranteed during data collection and analysis.

### 2.5. Sample Size Calculation

The expected stroke frequency among the general population was set at 3.9% based on previous research [24]. The CDC’s Epi Info version 7.2.4 for population surveys computed a minimal sample of 58 stroke survivors for post-stroke analyses. For the SS-QOL-17 validation, the participants-to-items ratio was required to be at least 10:1 [31]. Therefore, a minimal sample size of 170 participants was required to validate the SS-QOL-17, and to allow for adequate power of statistical analysis with 95% confidence level.

### 2.6. Statistical Analysis

The IBM Statistical Package for Social Sciences (IBM SPSS) version 26.0 was used to analyze data. Descriptive statistics were used to evaluate the sociodemographic, socioeconomic, and clinical characteristics. Continuous variables were expressed by their means (±standard deviation, SD), and categorical variables were expressed by their frequencies and percentages.

The SS-QOL-17 structure was validated by factor analysis using principal component analysis (PCA) with rotated matrix. The Kaiser–Meyer–Olkin (KMO) measure of sampling adequacy and Bartlett’s test of sphericity were confirmed to be adequate. Extracted factors from the final scale had Eigen values greater than one, and were used to generate the SS-QOL-17 total score. Pearson correlation was used to determine the correlation of each item of the scale with the entire scale. Cronbach’s alpha was used to measure internal consistency and reliability of the scale. The sensitivity and specificity of the scale were determined by ROC curve analysis, with optimal cut-off point determined by J-index.

The bivariate analysis taking the SS-QOL-17 score as the dependent variable involved Pearson correlation, independent sample T test, and one-way ANOVA. The Shapiro–Wilk test and histogram analysis were used to confirm the normal distribution of the SS-QOL-17 score.

Thereafter, a multivariable linear regression was performed to determine predictors of post-stroke QOL and exclude potential confounding. Three models were performed, taking the SS-QOL-17 score as the dependent variable and variables with *p*-values less than 0.2 in the bivariate analysis as independent variables. The first model presented the socioeconomic characteristics, whereas the second model presented the sociodemographic characteristics of the patients. In the third model, the therapeutic characteristics were added to the significant variables in the first and second models. The results were reported as unadjusted beta and 95% confidence interval (CI). The level of significance was set at a *p*-value < 0.05 with an acceptable margin of error = 5%.

## 3. Results

### 3.1. Sociodemographic and Socioeconomic Characteristics

A total of 172 stroke survivors were included, among which 61.6% were males, 70.9% were married, and 45.1% had three to four children. For the level of education, 46.5% had a school level, 43.0% were unemployed, and 27.9% had a monthly household income between LBP 2,000,000 and 3,500,000. The patients had a mean age of 62.67 (±13.38) and a mean IFDFW score of 39.31 (±19.53). The sociodemographic and socioeconomic characteristics of the patients are reported in Table 1.

### 3.2. Clinical Characteristics

The majority of stroke survivors (83.7%) had experienced one stroke only, 66.2% were diagnosed during the past one to five years, and 79.1% of the strokes were ischemic. The mean number of used medications and LMAS-14 score were 6.23 (±3.10) and 34.92 (±8.78), respectively. For the stroke outcomes, around three-fourths of the patients (76.7%) had poor prognosis on mRS and 46.5% had severe-to-total dependency on BI. Table 2 shows the full clinical characteristics of the patients.

### 3.3. Validation of the SS-QOL-17

#### 3.3.1. Factor Analysis

Factor analysis was performed to validate the structure of the SS-QOL-17 for post-stroke QOL. All 17 items of the scale could be extracted with Promax rotation. No variables had low factor loading (<0.3), low communality (<0.3), or over correlation with each other (r > 0.9). The KMO measure of sampling adequacy was 0.894 with a significant Bartlett’s test of sphericity (*p* < 0.001).

The scale converged on a solution of three factors with Eigenvalues more than one, explaining 67.85% of the total variance. Factor 1 of the scale included six items representing functional factors, Factor 2 included nine items representing psychosocial factors, and Factor 3 included two items representing therapeutic factors. Table 3 shows the rotated matrix of the SS-QOL-17.

#### 3.3.2. Validity Measures

The SS-QOL-17 had a high Cronbach’s alpha of 0.903 for assessing QOL among stroke survivors. All items of the scale significantly correlated with the full scale with *p* < 0.001. The Pearson correlation coefficients ranged from 0.311 to 0.761. Table 4 reports the correlation of the SS-QOL-17 items with the full scale.

**Figure 1:**
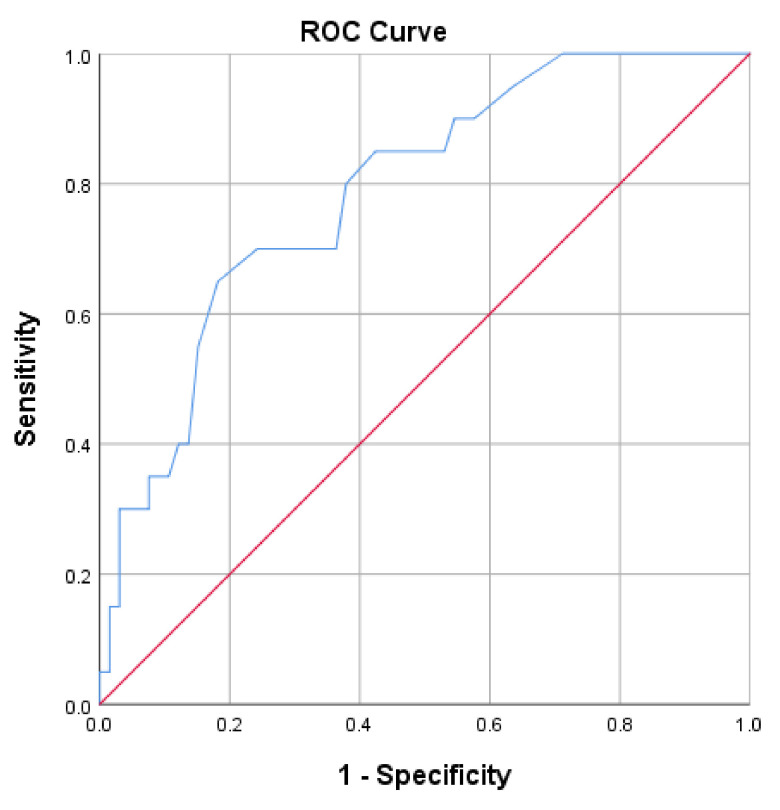
ROC curve of the SS-QOL-17. Stroke survivors with good prognosis on mRS were analyzed. Area under the curve = 0.787; 95% CI 0.712–0.863 (*p* < 0.001). At value = 44.00, sensitivity = 70.00% and specificity = 75.80%.

The SS-QOL-17 had a mean of 40.56 (±12.31), with higher values indicating better QOL. The ROC curve analysis revealed an optimal cut-off point of better QOL at 44.00 with 70.0% sensitivity and 75.8% specificity. The area under the curve was 0.787; 95% CI 0.712–0.863 (*p* < 0.001). Figure 1 presents the ROC curve of the SS-QOL-17 comparing stroke survivors with good prognosis on mRS to stroke survivors with poor prognosis.

### 3.4. Bivariate Analysis of Post-Stroke QOL

The bivariate analysis of the socioeconomic characteristics of the patients showed a significant positive correlation between the SS-QOL-17 score and the IFDFW score (r = 0.265, *p* < 0.001). The mean of the SS-QOL-17 score was also significantly different between the different categories of monthly household income (*p* = 0.001).

The bivariate analysis of the sociodemographic characteristics of the patients showed significantly different means of the SS-QOL-17 score between the different levels of education (*p* = 0.007), employment status (*p* = 0.009), and the presence and type of medical coverage/insurance (*p* < 0.001). There was also a significant negative correlation between the SS-QOL-17 score and age (r = −0.161, *p* = 0.035).

The bivariate analysis of the pharmacotherapeutic characteristics of the patients showed a significant negative correlation between the SS-QOL-17 score and the number of prescribed medications (r = −0.343, *p* < 0.001). However, there was a significant positive correlation between the SS-QOL-17 and LMAS-14 scores (r = 0.344, *p* < 0.001). The bivariate analysis of the socioeconomic, sociodemographic, and therapeutic characteristics of the patients is shown in Table 5.

### 3.5. Predictors of Post-Stroke QOL

Three multivariable linear regression models were performed, taking the SS-QOL-17 as the dependent variable. The first model included the socioeconomic characteristics of the patients as independent variables. The SS-QOL-17 score was significantly higher among stroke survivors, with better financial wellbeing reflected by the IFDFW score (Beta 0.093, *p* = 0.001). A significant negative association was also found between the SS-QOL-17 score and the monthly household income. Patients with a monthly household income of LBP 5,000,000 and less had significantly lower SS-QOL-17 scores compared to patients with a monthly household income greater than LBP 8,000,000.

The second model included the sociodemographic characteristics of the patients as independent variables. A significantly lower SS-QOL-17 score was associated with older age (beta −0.117, *p* = 0.014), being illiterate compared to having a university level of education (beta −6.428, *p* < 0.001), and being unemployed compared to being employed (beta −6.170, *p* < 0.001).

The third model included the therapeutics characteristics of the patients in addition to significant variables from the first and second models as independent variables. A significantly lower SS-QOL-17 score was associated with higher number of prescribed medications (Beta −1.148, *p* < 0.001), whereas a significantly higher SS-QOL-17 score was associated with a higher score of LMAS-14 (Beta 0.305, *p* = 0.004). The significant association between the SS-QOL-17 score and financial wellbeing was retained in the model. The multivariable linear regression, taking the SS-QOL-17 score as the dependent variable is reported in Table 6.

## 4. Discussion

This study constructed and validated the novel 17-item stroke-specific QOL scale (SS-QOL-17) with the aim to provide a well-balanced wide-ranging short tool to measure the QOL of stroke survivors. Our findings impart initial evidence to support the scale’s validity and reliability for this assessment. The scale demonstrated very good psychometric properties and internal consistency. Therefore, it is suggested for use in stroke research and practice. The post-stroke QOL was significantly predicted by the socioeconomic characteristics linked to financial wellbeing and monthly household income, and by the sociodemographic characteristics involving age, level of education, and employment. The post-stroke QOL was also predicted by the therapeutic characteristics relating to the number of prescribed pharmacotherapies and medication adherence.

The current study was able to develop the SS-QOL-17, a novel 17-item scale to measure the HRQOL after a stroke. The scale was constructed in reference to the existing ones but with enhanced balance between depicting a wider QOL profile and offering an abbreviated utility measure for simple administration to stroke survivors. Existing scales appear to have adequate reliability and internal consistency in our sample. Nevertheless, the practical use of those scales is restricted by their overlength or limited breadths of assessment. Hence, it was crucial to develop a novel short and simple scale that does not require much time to administer to stroke patients, yet is well-balanced enough to integrate a wide range of factors to illustrate the functional, psychosocial, and therapeutic dimensions. The SS-QOL-17 items were carefully adapted to combine the aspects of HRQOL linked to feeding, bathing, grooming, dressing, toileting, mobility, forgetfulness and unwillingness to take prescribed therapies, level of energy, engagement with family, speech difficulties, depressed mood and irritability, social engagement, cognition, and general performance difficulties.

Our findings on the psychometric properties showed that the SS-QOL-17 is reliable, as indicated by the Cronbach’s alpha value for the whole scale [32]. The SS-QOL-17 items were divided by factor analysis into three factors. Factor 1 (functional) was linked to the level of dependence in performing daily activities, Factor 2 (therapeutic) was linked to forgetfulness and willingness to comply with treatment, and Factor 3 (psychosocial) was linked to stroke sequelae on social and family roles, mood and cognition, and energy and general performance. The psychosocial domain was previously suggested by the existing 12-item version of the SS-QOL (SS-QOL-12), which adapted a selected 12 items from the long 49-item SS-QOL divided over two domains (physical and psychosocial) [22]. A major limitation of the existing SS-QOL-12 is that it adapted a theoretical domain division based on the long scale without determining the structure validity of the abbreviated 12-item scale [21]. Moreover, the deduction of 37 items from the original scale, without compensation, compromised functional measures of specific daily living activities. Thus, the existing SS-QOL-12 is inadequate to measure the HRQOL aspects that are directly linked to performing specific daily functions.

The internal consistency of the novel SS-QOL-17 was found to be good in comparison to the earlier versions of the SS-QOL. In fact, the Cronbach’s alpha of the SS-QOL-17 was 0.903, comparable to that of the 49-item SSQOL-A (0.979) among the same sample, and even higher than that determined for the SS-QOL-12 (0.850) [22]. In previous validation studies, the reliability assessment of the of the 49-item SS-QOL versions determined Cronbach’s alpha values ranging from 0.810 to 0.970 [33,34,35,36]. Although the correlation of the SS-QOL-17 items with the full scale was variable ranging from 0.311 to 0.761. This variation was expected because the scale incorporated items from three different post-stroke QOL dimensions. Furthermore, the reproducibility of the SS-QOL-17 was confirmed by the highly significant correlation of each item of the scale with the full scale.

The construct validity of the SS-QOL-17 was verified by computing the sensitivity and specificity of the scale. The SS-QOL-17 appears to have a good sensitivity and specificity for determining the stroke-related QOL [37]. Nevertheless, it was not possible to compare this sensitivity and specificity with existing literature because of the scale’s novelty and since the sensitivity and specificity of the existing versions of the SS-QOL were not previously determined. Further research is suggested in this context to confirm the construct validity of this new scale in other populations and languages.

This study also evaluated the role of socioeconomic, sociodemographic, and therapeutic characteristics of patients in predicting post-stroke QOL. We found a significant correlation between financial wellbeing and a better QOL. Although there is scarcity of data in the literature about the direct correlation between financial wellbeing and stroke outcomes, previous studies established a significant correlation between monthly income and QOL [38,39]. Those findings are supported by our results as we determined a significantly lower score of SS-QOL-17 among patients with lower monthly household income. Therefore, the financial aspects appear to be as critical as health-related factors in shaping the QOL of stroke survivors.

The assessment of sociodemographic characteristics showed a significant correlation between QOL and age, level of education, and employment. Our results are consistent with other findings that determined a reduced QOL with older age, lower level of education, and unemployment [39,40,41]. Indeed, an older age is associated with higher stroke morbidity, disability, and polytherapy, all of which could impair the HRQOL [24]. Moreover, a lower level of education is reportedly associated with increased risk of stroke complications and recurrences [42]. This can be explained as a result of lower adherence to stroke therapy [24]. The level of education could be also an important predictor of employment and better socioeconomic status, and thus overall QOL. Although this three-dimensional association was not established in stroke research, employed stroke survivors are generally determined to be in a better financial situation and therefore a better QOL [43]. Nonetheless, the correlation between employment and QOL might not be exclusively explained by the socioeconomic status, as it was suggested that returning to work after a stroke could accelerate recovery of the functional disability and improve QOL [39].

Our findings on the therapeutic characteristics showed a significant correlation between QOL and the number of prescribed medications and therapeutic adherence. Patients with higher number of prescribed medications had reduced QOL, whilst better adherence to medications was associated with better QOL. These results are consistent with previous research that reported an important association between poor stroke outcomes and the number of prescribed therapies [24]. The current study adds to the literature that therapeutic adherence could improve stroke outcomes and QOL. Indeed, therapeutic adherence after a stroke is essential to achieve the targeted goals of treatment and obtain the greatest long-term outcomes [44,45]. The current findings provide additional evidence to support the therapeutic factor criterion validity in measuring stroke-related QOL.

### 4.1. Practical Implications

Stroke survivors often experience a considerable reduction in their HRQOL. The existing stroke-specific QOL measuring tools are limited by several drawbacks linked to the overlength and inconsistent structure validity of the long 49-item versions of the SS-QOL, and to the compromised breadths of assessment of the shorter 12-item version. The long 49-item SS-QOL provides a comprehensive assessment for 12 post-stroke QOL domains pertaining to social role, mobility, energy, language, self-care, mood, personality, thinking, upper extremity function, family role, vision, and work/productivity. The 49-item scale is overly long for routine use in stroke research and clinical practice. Moreover, the construct validity of the scale is inconsistent as previous research produced one-, two-, four-, eight-, and twelve-domain solutions for its structure. On the other hand, the SS-QOL-12 is an abbreviated form of the long SS-QOL. It assesses two domains of the post-stroke QOL relating to physical and psychosocial factors. Although the scale is short and practically more tempting, it does not assess functions for daily living such as feeding, dressing, grooming, and toileting. Similarly, it does not assess therapeutic adherence, irritability, and family engagement.

The findings of the current research produce a short yet comprehensive measuring tool to support stroke service and research systems. The SS-QOL-17 appears to be an excellent measuring tool of stroke-related QOL, and thus it is highly recommended for stroke research and practice. This novel scale enables one to evaluate the functional performance for daily living as well as the therapeutic and psychosocial features. The SS-QOL-17 assesses post-stroke QOL relating to daily performance on feeding, bathing, grooming, dressing, toileting, and mobility. It also assesses energy, family engagement, speech troubles, future discouragement, mood, cognition, social engagement, general performance, and therapeutic adherence. Similar to other health-related scales that were developed in Lebanon to measure health-related features, and thus were entitled “Lebanese”, this Arabic version of the SS-QOL-17 will be also labeled as the Lebanese Post-Stroke Scale (LPSS-17). Future research is still recommended to confirm the validity of this scale within other cultures and languages. Additionally, our findings on the correlation between the socioeconomic (financial), sociodemographic (age, employment, and level of education), and therapeutic (number of prescribed therapies and medication adherence) factors and stroke-related QOL are prominent, and warrant comprehensive rehabilitation plans that should integrate the functional, therapeutic, and psychosocial improvements along with the socioeconomic and sociodemographic indicators in order to heighten the QOL of stroke survivors.

### 4.2. Strengths and Limitations

Although stroke prevalence is quite low in Lebanon, this study included a sufficient sample size that ensured adequate power for all statistical analyses. The sample size was comparable to previous research on the QOL of stroke survivors in Europe [46], and even greater compared to the sample size of another study from the Middle East [47]. The sample also included patients from all over the Lebanese districts, which minimized the risk of selection bias that could be attributed to different QOL indicators of patients from diverse societal backgrounds. Moreover, the current sample was coherent with the usual post-stroke trend of having more stroke survivors with poor prognosis [48], and thus allowed to assess the QOL of these patients. However, several limitations should be acknowledged. First, there is no authentic gold standard for selecting a criterion validity measure for post-stroke QOL. The present study selected the mRS to examine the validity of the SS-QOL-17, which might be more linked to the functional than psychosocial QOL domain. Future work may utilize generic QOL criterion measures to reflect more on the psychosocial domain and provide additional evidence of validity. Second, there were two different methods of data collection in this study (phone calls and face-to-face). This could have also been associated with a potential risk of information bias, although it is believed that this risk is minimal because data reporting involved proclaimed responses rather than observing patient reactions. Third, the study design was cross-sectional, which cannot determine temporality and confirm the causality of socioeconomic, sociodemographic, and therapeutic factors. Prospective studies are still recommended to follow up on stroke outcomes and illustrate further evidence on the QOL predictors. However, it is believed that such kinds of studies may still carry a possible risk of information bias in consequence to the negative and positive changes of stroke outcomes over time. Finally, our multivariable analysis of post-stroke QOL did not include independent mental health predictors; thus, residual mental health confounding cannot be excluded. Future work will evaluate the impact of the different categories of mental health on the QOL of stroke survivors.

## 5. Conclusions

The SS-QOL-17 is a valid and reliable tool with promising psychometric properties to assess the QOL of stroke survivors. It is projected to be useful in clinical practice and research settings to measure stroke sequelae and evaluate therapeutic and rehabilitation outcomes. The SS-QOL-17 appears practical and beneficial to determine the HRQOL after a stroke and to identify patients who should receive greater attention to their management plan and rehabilitation strategy, to possibly explore areas for improving their QOL. Further studies are recommended to provide additional evidence on the validity of the SS-QOL-17 in Arabic-speaking nations and to confirm its validation in other languages and populations. This study determined that post-stroke QOL is significantly predicted by financial wellbeing; sociodemographic factors involving age, education level, and employment; and therapeutic factors involving number of prescribed therapies and medication adherence. It is definitely warranted to recognize and address lower levels of QOL with the aim to maximize care and support to improve stroke outcomes.

## Figures and Tables

**Table 1:** Sociodemographic and socioeconomic characteristics of the patients.

Variable	Category	Stroke Survivors (n = 172)	
		Mean or Frequency	SD or %
Age		62.67	13.38
Gender	Male Female	106 66	61.6 38.4
BMI *		26.81	3.40
Area of residence	Beirut Bekaa Mount Lebanon North South	78 12 40 14 28	45.30 7.0 23.30 8.10 16.30
Smoking status	Current smoker Ex-smoker Non-smoker	62 66 44	36.0 38.4 25.6
Alcohol consumption	In the past, not anymore No Yes, currently	36 122 14	20.9 70.9 8.1
Marital status	Divorced Married Single Widowed	8 122 22 20	4.7 70.9 12.8 11.6
Number of children (N = 142)	1 to 2 3 to 4 More than 4	32 64 46	22.5 45.1 32.4
Level of education	Illiterate School level University level	48 80 44	27.9 46.5 25.6
Employment	Employed Retired Unemployed	46 52 74	26.7 30.2 43.0
Household income	LBP < 2,000,000 LBP 2,000,000–3,500,000 LBP 3,500,000–5,000,000 LBP 5,000,000–6,500,000 LBP 6,500,000–8,000,000 LBP > 8,000,000	40 48 32 22 6 24	23.3 27.9 18.6 12.8 3.5 14.0
Living:	Alone With family (nuclear or extended)	16 156	9.3 90.7
IFDFW * score		39.31	19.53

* BMI: body mass index; IFDFW: InCharge Financial Distress/Financial Well-Being Scale.

**Table 2:** Clinical characteristics of the patients.

Variable	Category	Stroke Survivors (n = 172)	
		Mean or Frequency	SD or %
Date of stroke diagnosis	Less than 1 year 1 to 5 years More than 5 years	34 114 24	19.8 66.2 14.0
Type of stroke	Hemorrhagic Ischemic	36 136	20.9 79.1
First or recurrent stroke	First Recurrent	144 28	83.7 16.3
Number of comorbidities		3.80	2.44
Number of medications		6.23	3.10
LMAS-14 * score		34.92	8.78
mRS *	Good prognosis Poor prognosis	40 132	23.3 76.7
BI * classes	Slight dependency Moderate dependency Severe dependency Total dependency	64 28 44 36	37.2 16.3 25.6 20.9

* LMAS-14: Lebanese Medication Adherence Scale; mRS: modified Rankin Scale; BI: Barthel Index.

**Table 3:** Promax-rotated matrix of the SS-QOL-17.

Factor	Items	Factor 1	Factor 2	Factor 3	Communalities
Toilet use	BI 7	0.970			0.875
Dressing	BI 4	0.945			0.865
Feeding	BI 1	0.928			0.816
Bathing	BI 2	0.881			0.818
Grooming	BI 3	0.869			0.750
Mobility	BI 9	0.841			0.777
My physical condition interfered with my social life	SS-QOL 35		0.840		0.706
I didn’t go out as often as I would like	SS-QOL 31		0.837		0.691
I felt tired most of the time	SS-QOL 1		0.817		0.642
I didn’t join in activities just for fun with my family	SS-QOL 4		0.777		0.644
I was irritable	SS-QOL 23		0.724		0.431
I was discouraged about my future	SS-QOL 18		0.697		0.493
Did you have trouble doing the work you used to do?	SS-QOL 49		0.657		0.632
It was hard for me to concentrate	SS-QOL 36		0.599		0.553
Did you have trouble speaking? For example, get stuck, stutter, stammer, or slur your words?	SS-QOL 7		0.440		0.487
Do you forget taking your medication?	LMAS 13			0.830	0.690
Do you stop taking your medications if they are expensive?	LMAS 14			0.823	0.666
*Percentage of variance explained*		44.47%	15.16%	8.22%	

Factor 1 = functional; Factor 2 = psychosocial; Factor 3 = therapeutic. Cronbach’s alpha for the SS-QOL-17 = 0.903. Total percentage of variance explained: 67.85%. Kaiser–Meyer–Olkin (KMO) = 0.894. Bartlett’s test of sphericity: *p* < 0.001.

**Table 4:** Pearson correlation of the SS-QOL-17 items with the full scale among stroke survivors.

SS-QOL-17 Item Number	Items	r *	*p* Value
1	Feeding	0.637	<0.001
2	Bathing	0.684	<0.001
3	Grooming	0.620	<0.001
4	Dressing	0.677	<0.001
5	Toilet use	0.655	<0.001
6	Mobility	0.711	<0.001
7	Do you forget taking your medication?	0.355	<0.001
8	Do you stop taking your medications if they are expensive?	0.311	<0.001
9	I felt tired most of the time.	0.714	<0.001
10	I didn’t join in activities just for fun with my family.	0.749	<0.001
11	Did you have trouble speaking? For example, get stuck, stutter, stammer, or slur your words?	0.706	<0.001
12	I was discouraged about my future.	0.599	<0.001
13	I was irritable.	0.520	<0.001
14	I didn’t go out as often as I would like.	0.754	<0.001
15	My physical condition interfered with my social life.	0.761	<0.001
16	It was hard for me to concentrate.	0.701	<0.001
17	Did you have trouble doing the work you used to do?	0.771	<0.001

* Pearson correlation coefficient.

**Table 5:** Bivariate analysis of the socioeconomic, sociodemographic, and therapeutic characteristics of the patients taking the SS-QOL-17 as the dependent variable.

Variable	Category	Mean	SD	*p* Value
Gender	Male	40.49	12.67	0.928
	Female	40.67	11.81	
Marital status	Divorced	35.25	8.43	0.612
	Married	41.1	12.3	
	Single	40.27	13.19	
	Widowed	39.7	12.94	
Number of children (N = 142)	1 to 2	38.38	11.69	0.385
	3 to 4	42.09	13.24	
	More than 4	40.43	11.97	
Level of education	Illiterate	38.08	11.59	0.007 ^a^
	School level	39.33	11.93	
	University level	45.5	12.65	
Employment	Employed	45.22	12.98	0.009 ^b^
	Unemployed	38.19	10.37	
	Retired	39.32	12.53	
Household income	LBP < 2,000,000	40.1	13.72	0.001 ^c^
	LBP 2,000,000–3,500,000	38.04	9.52	
	LBP 3,500,000–5,000,000	39.88	11.18	
	LBP 5,000,000–6,500,000	36.82	10.35	
	LBP 6,500,000–8,000,000	42	10.32	
	LBP > 8,000,000	50.33	14.33	
Medical coverage/insurance	No	36.64	11.99	<0.001 ^d^
	Yes, NSSF *	37.17	9.61	
	Yes, private medical insurance or private mutual fund (with or without NSSF *)	47.9	13.14	
	Yes, coverage through the public or military sector (other than NSSF *)	41.82	11.04	
Living:	Alone	39.63	14.01	0.751
	With family (nuclear or extended)	40.65	12.17	
Medications coverage by third-party payers	No	40.63	13.93	0.996
	Yes, completely	40.6	12.72	
	Yes, partially	40.46	10.17	
Financial difficulties to obtain medications	No	42.17	12.57	0.06
	Yes, mild difficulty	43.96	12.44	
	Yes, severe difficulty	38.85	13.36	
	Yes, moderate difficulty	38.2	10.19	
Obtaining medications from outside the country due to their unavailability in Lebanon	No	38.15	12.41	0.088
	Yes, sometime	41.75	10.33	
	Yes, always	42.5	7.15	
	Yes, most of the time	44.91	16.89	
**Variable**		Correlation Coefficient **		***p* Value**
Age		−0.161		0.035
IFDFW * score		0.265		<0.001
Number of medications		−0.343		<0.001
LMAS-14 * score		0.344		<0.001

* NSSF: National Social Security Fund; IFDFW: InCharge Financial Distress/Financial Well-Being Scale, LMAS-14: Lebanese Medication Adherence Scale. ** Pearson correlation coefficient. ^a^ Post hoc analysis showed significant difference between “Illiterate” and “University level” (*p* = 0.011); and “School level” and “University level” (*p* = 0.021). ^b^ Post hoc analysis showed significant difference between “Employed” and “Unemployed” (*p* = 0.013); and “Employed” and “Retired” (*p* = 0.030). ^c^ Post hoc analysis showed significant difference between “LBP > 8,000,000” and “LBP <2,000,000” (*p* = 0.014), “LBP 2,000,000–3,500,000” (*p* = 0.001), “LBP 3,500,000–5,000,000” (*p* = 0.018), and “LBP 5,000,000–6,500,000” (*p* = 0.002). ^d^ Post hoc analysis showed significant difference between “Yes, private medical insurance or private mutual fund (with or without NSSF)” and “No” (*p* < 0.001); and “Yes, private medical insurance or private mutual fund (with or without NSSF) and “Yes, NSSF” (*p* < 0.001).

**Table 6:** Multivariable linear regression taking the SS-QOL-17 as the dependent variable.

Model 1 Including Socioeconomic Characteristics *					
Variable	Unstandardized Beta	Standardized Beta	*p* Value	95% Confidence Interval	
				Lower	Upper
**IFDFW** **^‡^ score**	0.093	0.142	0.001	0.037	0.149
**Household income (reference: LBP > 8,000,000)** LBP <2,000,000 LBP 2,000,000–3,500,000 LBP 3,500,000–5,000,000 LBP 5,000,000–6,500,000	−4.683 −5.476 −3.903 −2.897	−0.137 −0.183 −0.118 −0.084	0.008 <0.001 0.018 0.088	−8.132 −8.54 −7.124 −6.225	−1.234 −2.412 −0.681 0.432
Model 2 Including Sociodemographic Characteristics **					
**Variable**	**Unstandardized Beta**	**Standardized Beta**	***p*** **Value**	**95% Confidence Interval**	
				**Lower**	**Upper**
**Age**	−0.117	−0.112	0.014	−0.209	−0.024
**Level of Education (Illiterate vs. University level)**	−6.428	−0.184	<0.001	−9.38	−3.477
**Employment (reference: Employed)** Unemployed Retired	−6.170 −2.142	−0.177 −0.083	<0.001 0.080	−9.543 −4.542	−2.797 0.258
Model 3 Including the Therapeutic Characteristics and Significant Variables in Models 1 and 2 ***					
**Variable**	**Unstandardized Beta**	**Standardized Beta**	***p*** **Value**	**95% Confidence Interval**	
				**Lower**	**Upper**
**Number of medications**	−1.148	−0.289	<0.001	−1.701	−0.595
**LMAS-14** **^‡^** **score**	0.305	0.217	0.004	0.099	0.511
**IFDFW** **^‡^** **score**	0.123	0.194	0.008	0.033	0.213

* Variables initially included in the model: financial difficulties to obtain medications; obtaining medications from outside the country due to their unavailability in Lebanon. ** Variables initially included in the model: availability of medical coverage or insurance. *** Variables initially included in the model: household income; age; level of education; employment. ^‡^ IFDFW Scale: InCharge Financial Distress/Financial Well-Being Scale; LMAS-14: Lebanese Medication Adherence Scale.

## Data Availability

The data presented in this study are available from the corresponding author on reasonable request.
